# Integrated transcriptome and metabolome analysis revealed that flavonoids enhanced the resistance of *Oryza sativa* against *Meloidogyne graminicola*

**DOI:** 10.3389/fpls.2023.1137299

**Published:** 2023-03-31

**Authors:** Lianhu Zhang, Songyan Li, Chonglei Shan, Yankun Liu, Yifan Zhang, Lifang Ye, Yachun Lin, Guihong Xiong, Jian Ma, Muhammad Adnan, Xugen Shi, Xiaotang Sun, Weigang Kuang, Ruqiang Cui

**Affiliations:** ^1^ College of Agronomy, Jiangxi Agricultural University, Nanchang, China; ^2^ Shenzhen Key Laboratory of Microbial Genetic Engineering, College of Life Sciences and Oceanography, Shenzhen University, Shenzhen, China; ^3^ Key Laboratory of Crop Physiology, Ecology and Genetic Breeding, Ministry of Education, Jiangxi Agricultural University, Nanchang, China

**Keywords:** transcriptome, metabolome, resistance of *Oryza sati*va, flavonoids, *Meloidogyne graminicola*

## Abstract

Rice is a crucial food crop worldwide, but its yield and quality are significantly affected by *Meloidogyne graminicola* is a root knot nematode. No rice variety is entirely immune to this nematode disease in agricultural production. Thus, the fundamental strategy to combat this disease is to utilize rice resistance genes. In this study, we conducted transcriptome and metabolome analyses on two rice varieties, ZH11 and IR64. The results indicated that ZH11 showed stronger resistance than IR64. Transcriptome analysis revealed that the change in gene expression in ZH11 was more substantial than that in IR64 after *M. graminicola* infection. Moreover, GO and KEGG enrichment analysis of the upregulated genes in ZH11 showed that they were primarily associated with rice cell wall construction, carbohydrate metabolism, and secondary metabolism relating to disease resistance, which effectively enhanced the resistance of ZH11. However, in rice IR64, the number of genes enriched in disease resistance pathways was significantly lower than that in ZH11, which further explained susceptibility to IR64. Metabolome analysis revealed that the metabolites detected in ZH11 were enriched in flavonoid metabolism and the pentose phosphate pathway, compared to IR64, after *M. graminicola* infection. The comprehensive analysis of transcriptome and metabolome data indicated that flavonoid metabolism plays a crucial role in rice resistance to *M. graminicola* infection. The content of kaempferin, apigenin, and quercetin in ZH11 significantly increased after *M. graminicola* infection, and the expression of genes involved in the synthetic pathway of flavonoids also significantly increased in ZH11. Our study provides theoretical guidance for the precise analysis of rice resistance and disease resistance breeding in further research.

## Introduction

Rice is a crucial food crop globally (Dimkpa et al., 2016), and it serves as the primary staple in our country (Khush, 2005; Matsuda, 2019). Ensuring safe rice production is vital for national food security. However, rice cultivation faces numerous challenges such as diseases, pests, and weeds, which severely limit yield and quality (Deb et al., 2021) (http://www.knowledgebank.irri.org). Therefore, strengthening the prevention and control of rice epidemics caused by diseases, pests, and weeds is a critical task.

In recent years, the rice root knot nematode, *Meloidogyne graminicola*, has emerged as a significant pathogen. It was first detected in Hainan Province, China, in 2001 (Honghai et al., 2001), and has since been reported in various rice-growing regions in southern China (Ju et al., 2020; Luo et al., 2020; Liu et al., 2021). This parasitic nematode disease has significantly decreased rice yield, making it one of the most severe nematodes in rice production (Mantelin et al., 2017). Therefore, understanding and mastering the development patterns of this disease can help achieve efficient disease prevention and control.

Currently, planting resistant rice varieties is considered the most effective way to manage rice root knot nematode disease (Trudgill, 1991). The main disease-resistant gene in rice that can resist *M. graminicola* is located in the 6 Mb region of the chromosome (Lahari et al., 2019); however, this gene has not yet been cloned successfully, indicating that there is still a long way to go in breeding rice varieties that are immune to *M. graminicola*. Therefore, it is crucial to continue efforts to clone resistance genes and screen existing seed resources for resistance to *M. graminicola*. Various studies have highlighted that different rice varieties exhibit varying levels of resistance to *M. graminicola*, with African wild rice showing high resistance, although it is not easily adaptable to Asian planting areas (Mattos et al., 2019). Therefore, it is more practical to pursue the planting of rice varieties resistant to *M. graminicola* in Asia, particularly for Chinese rice breeding. In this regard, Dimkpa identified two varieties with high resistance, “KPM” and “LD24”, from 332 Asian rice germplasm resources (Dimkpa et al., 2016), while Zhan found that the rice varieties ZH11, “Shenliangyou1”, and “Clingyou4488” were resistant to *M. graminicola* in 136 Chinese rice germplasm resource banks (Zhan et al., 2018). Despite these findings, there is still no rice variety that is immune to *M. graminicola*, and therefore, more research is required to explore potential disease resistance genes and their mechanisms of resistance.

The biosynthesis of flavonoids is a significant downstream component of phenylpropanoid metabolism that can increase plant resistance. For instance, research has demonstrated that *Zanthoxylum bungeanum* plants that are resistant to stem canker have a considerably higher flavonoid content than susceptible plants (Li et al., 2021). Increasing the flavonoid content in tomatoes has been found to improve resistance to powdery mildew and reduce the spread of *tomato yellow leaf curl virus* (Yao et al., 2019). Additionally, flavonoids have an essential role in the interaction between plants and nematodes. When nematodes infect plant roots, flavonoids are induced, which can impact feeding site development (Chin et al., 2018). Therefore, it is crucial to study the biosynthesis and metabolism pathways of flavonoids to comprehend the mechanism of rice resistance, which has practical significance.

Our study involved infecting the rice varieties ZH11 and IR64 with *M. graminicola*, followed by an analysis of the differences in gene expression and metabolite biosynthesis in their transcriptome and metabolome. We found that flavonoid metabolism played a crucial role in enhancing the rice response to *M. graminicola* infection. These results provide a theoretical foundation for the future development of rice varieties with enhanced resistance to *M. graminicola* through breeding.

## Results

### Resistance of different rice varieties against *M. graminicola* infection

**Figure 1:**
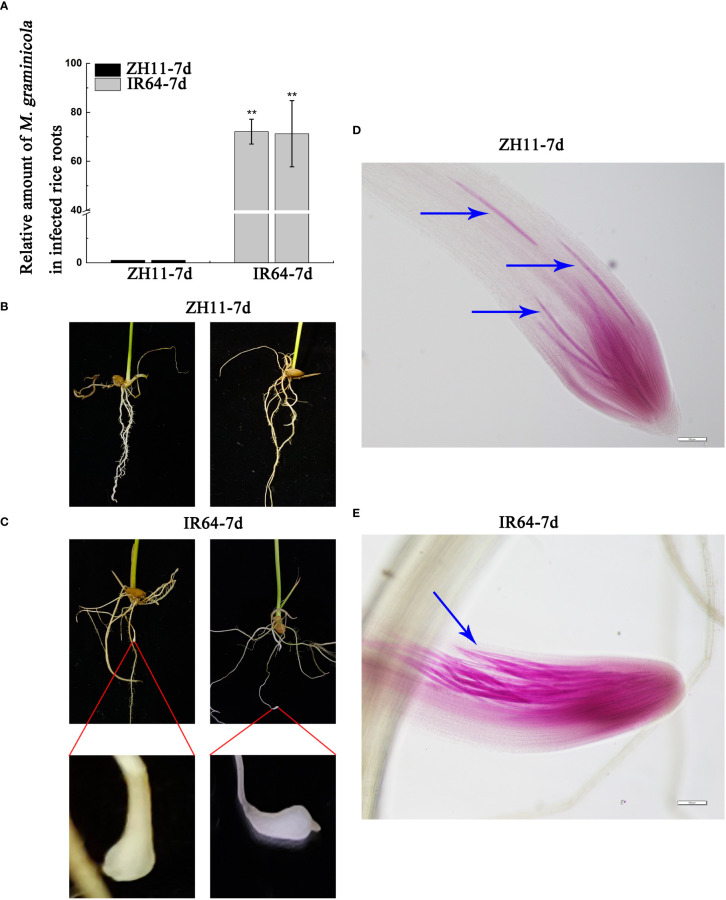
The relative amount of *M. graminicola* in infected roots of ZH11 and IR64. **(A)** shows the RT-qPCR results of the amount of *M. graminicola* in the infected roots of ZH11 and IR64. **(B, C)** show *M. graminicola* infection after 7 days; the roots of ZH11 had no root knots, but the roots of IR64 produced root knots, which were enlarged for the clarity of the display. **(D, E)** show that the roots of ZH11 and IR64 are infected by *M. graminicola*.

Previous studies have shown that rice variety ZH11 has strong resistance to *M. graminicola* infection, while rice variety IR64 has weak resistance (Phan et al., 2018). To verify this result, we conducted some experiments again. We infected the rice roots with nematodes for 7 days and found that the nematode infection amount in ZH11 rice roots was significantly lower than that in IR64 rice roots. Moreover, root knots were observed in IR64 roots, while ZH11 roots had no knots, which confirms the reliability of the above conclusions (**Figure 1**). To explain the difference between the disease resistance of ZH11 and IR64, we carried out transcriptome and metabolome analyses of rice root tissues infected with *M. graminicola*.

### Identification of DEGs of *O. sativa roots* in response to *M. graminicola* infection

To compare the difference in disease resistance of the rice varieties, we divided them into four different groups: ZH11-ck and IR64-ck, which are grown under normal conditions and without any sort of nematode infection; and ZH11-7d and IR64-7d, which represent rice roots that have been infected with *M. graminicola* for 7 days. Each group had three biological replicates to ensure the accuracy of the results and avoid any sort of miscalculation. Transcriptome data information for 12 samples is described in **Table S1** and the “Quality Analysis of Transcriptome Data” in the *Materials and methods* section. The correlation analysis of gene expression levels among the samples showed that the samples had high consistency in one group, which ensured the reliability of subsequent analyses (**Figure S1**). The gene expression profiles of 12 samples were analyzed by PCA. The results showed that the significant difference between the infected rice roots and the healthy rice roots was 26.90% from PC1, and the difference between rice varieties was mainly 21.82% from PC2 (**Figure S2**).

**Figure 2:**
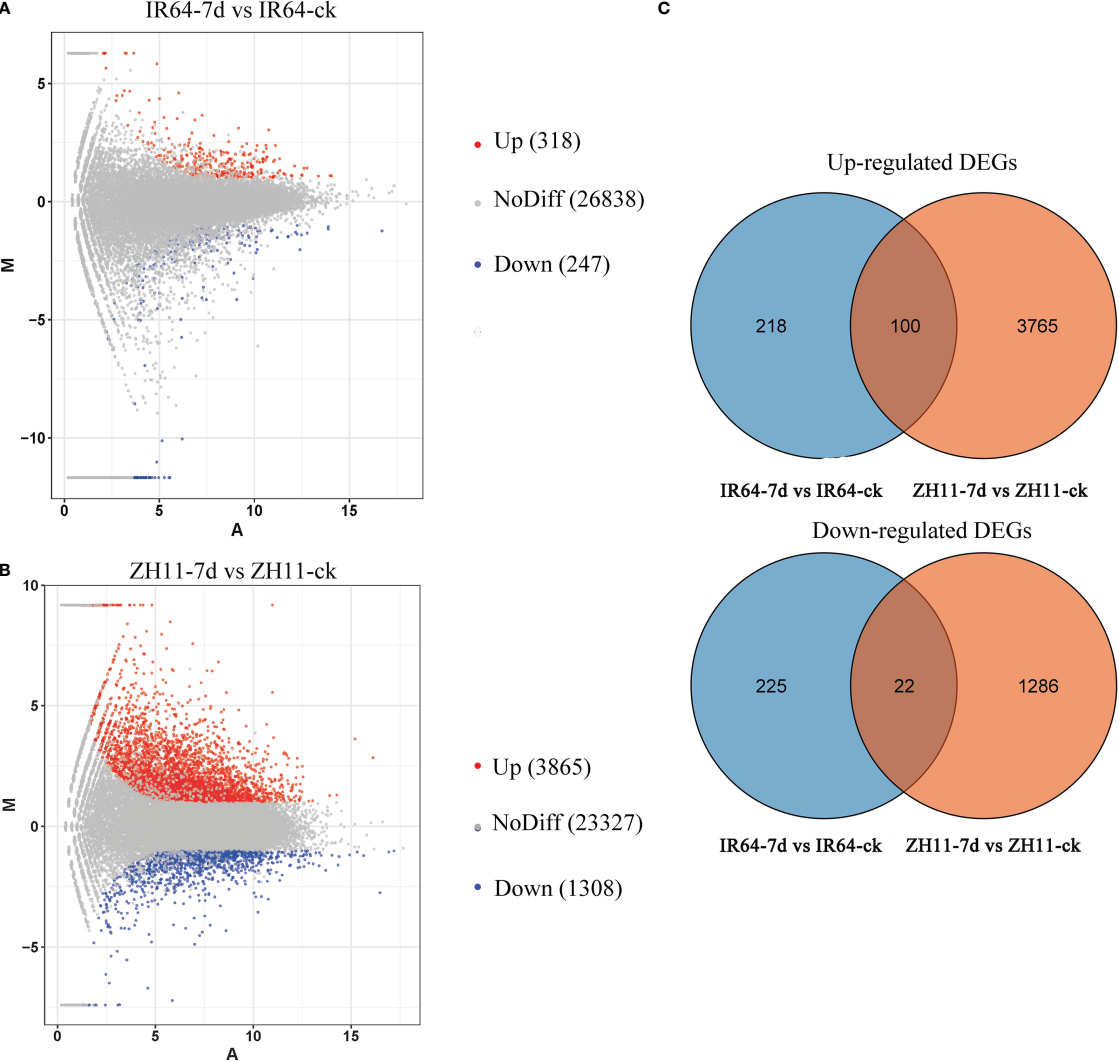
Analysis of transcriptome data from the rice roots for four groups: IR64-ck, IR64-7d, ZH11-ck, and ZH11-7d. **(A)** MA plots for pairwise expression analysis of transcriptome data of rice roots between IR64-ck and IR64-7d; **(B)** MA plots for pairwise expression analysis of transcriptome data of rice roots between ZH11-ck and ZH11-7d; **(C)** Venn diagram showing the upregulated DEGs and downregulated DEGs in comparison groups ZH11-7d vs ZH11-ck and IR64-7d vs IR64-ck.

By comparing the transcriptome data of four groups, IR64-ck, IR64-7d, ZH11-ck, and ZH11-7d, we found that IR64-7d has 565 differentially expressed genes (DEGs) compared with IR64-ck, 318 upregulated and 247 downregulated genes (**Figure 2A**, **Table S2**, **Sheets 1, 2**); while ZH11-7d has 5173 DEGs compared with ZH11-ck, 3865 upregulated and 1,308 downregulated genes (**Figure 2B**, **Table S2**, **Sheets 3, 4**). These results showed that gene expression in the roots of resistant variety ZH11 and susceptible variety IR64 has significantly changed after *M. graminicola* infection. Moreover, the Venn diagram showed that there were 100 upregulated unigenes and 22 downregulated unigenes in the comparison groups ZH11-7d vs ZH11-ck and IR64-7d vs IR64-ck. A total of 3,765 unique upregulated genes were found in the comparison group ZH11-7d vs ZH11-ck, which may be related to the resistance of rice ZH11 against *M. graminicola* infection; however, 218 unique upregulated genes were found in the comparison group IR64-7d vs IR64-ck, which may assist in *M. graminicola* infection in the susceptible variety IR64 (**Figure 2C**).

### Analysis of DEGs in *O. sativa roots* in response to *M. graminicola* infection

**Figure 3:**
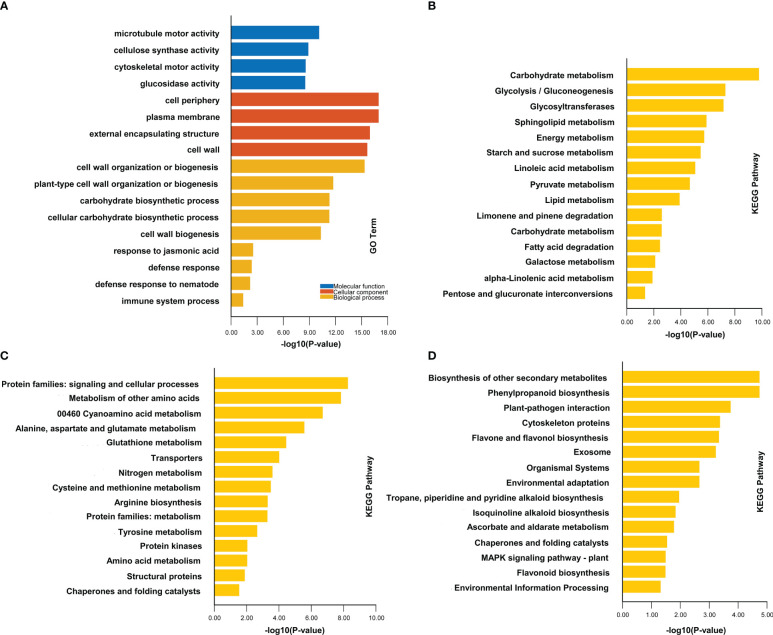
GO and KEGG analyses of DEGs in comparison groups ZH11-7d vs ZH11-ck. **(A)** indicates the GO enrichment of DEGs in comparison group ZH11-7d vs ZH11-ck. **(B)** indicates the KEGG enrichment of DEGs in carbohydrate metabolism in comparison group ZH11-7d vs ZH11-ck. **(C)** indicates the KEGG enrichment of DEGs in protein metabolism in comparison group ZH11-7d vs ZH11-ck. **(D)** indicates the KEGG enrichment of DEGs in plant resistance metabolism in comparison group ZH11-7d vs ZH11-ck.

To understand the mechanism of rice resistance to *M. graminicola* infection, we used GO and KEGG analysis to make an in-depth functional exploration of these DEGs (**Table S2**, **Sheets 6–10**). In the comparison group ZH11-7d vs ZH11-ck, 3,765 unique upregulated genes may be related to the disease resistance against *M. graminicola* infection. Therefore, we analyzed the function of these upregulated genes. The results of GO enrichment of upregulated DEGs suggest that these genes are mainly involved in cell wall biosynthesis, such as cell wall organization or biogenesis (GO:0071554), plant-type cell wall organization or biogenesis (GO:0071669) and cell wall biogenesis (GO:0042546). Additionally, genes enriched in the carbohydrate biosynthetic process (GO:0016051) and cellular carbohydrate biosynthetic process (GO:0034637) are also related to the cell wall synthetic process because a lot of carbohydrates are required for cell wall synthesis. Moreover, genes related to disease resistance were enriched in responses to jasmonic acid (GO:0009753), defense response (GO:0006952), and immune system processes (GO:0002376) (**Figure 3A**, **Table S2**, **Sheet 9**). Accordingly, we found that KEGG enrichment was mainly in three aspects. The first one was carbohydrate metabolism, which was directly related to the synthesis of carbohydrate compounds such as cellulose, hemicellulose, and pectin in plant cell walls. The second aspect was amino acid metabolism, which worked together with the third aspect of the plant resistance pathway, participating in plant secondary metabolic processes such as phenylpropanoid biosynthesis (ko00940), flavonoids metabolism (ko00941 and ko00944), and plant signal transduction processes such as protein kinases (ko01001), MAPK pathway (ko04016), and plant pathogen interaction process (ko04626) (**Figures 3B–D**, **Table S3**, **Sheet 3**). These three aspects greatly improved the resistance of ZH11 and effectively resisted the infection of nematodes.

Although, in the comparison group IR64-7d vs IR64-ck, we found that the upregulated genes were also enriched in some resistance pathways, such as response to jasmonic acid (GO:0009753), response to hormone (GO:0009725), regulation of jasmonic acid-mediated signaling pathway (GO:2000022), and defense response (GO:0006952); the number of genes enriched in these pathways was significantly less than that of ZH11 (**Figures S3A**, **S4**). Moreover, the upregulated genes did not participate in the cell wall biosynthesis pathway or the biosynthesis of some important secondary metabolites in rice (**Figure S3**, **Tables S2**, **S3**). Therefore, IR64 was susceptible to *M. graminicola* infection as compared to ZH11.

### Metabolome analysis of *O. sativa* roots in response to *M. graminicola* infection

**Figure 4:**
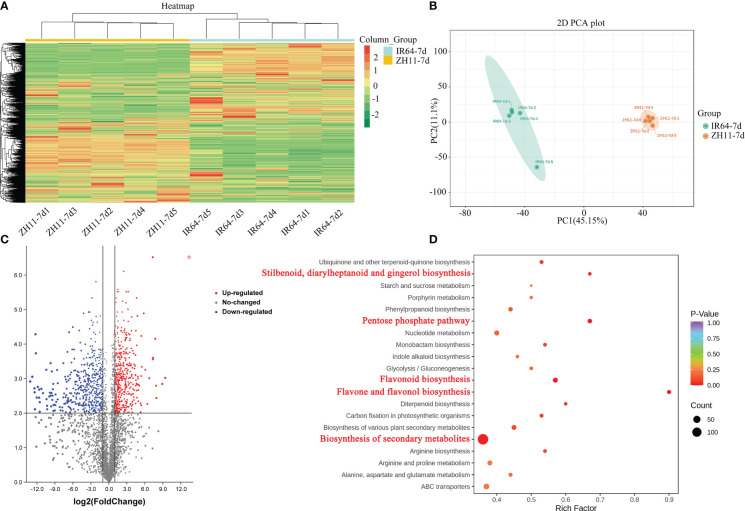
Overview of the widely targeted metabolome analysis of rice roots from ZH11 and IR64 in response to *M. graminicola* infection. **(A)** Heatmap visualization of metabolites. The content of each metabolite was normalized to complete linkage hierarchical clustering. Each example was visualized in a single column, and each metabolite is represented by a single row. Red indicated high abundance metabolites, and green indicated low abundance metabolites. **(B)** PCA analysis of metabolites showed that the difference in metabolites was mainly due to the difference in rice varieties. **(C)** The volcano plot indicates the differentially accumulated metabolites (DAMs) between ZH11 and IR64 roots infected by *M. graminicola*. **(D)** KEGG enrichment pathways of DAMs. The marked red KEGG terms highlight the significant enrichment metabolism pathways.

A metabolome analysis was conducted on root samples collected from rice varieties IR64 and ZH11, which are respectively susceptible and resistant to *M. graminicola* infection, at the 7th day post-infection (dpi). The samples were denoted as IR64-7d and ZH11-7d. The analysis resulted in the identification of 4,941 differentially accumulated metabolites (DAMs) across all samples, as demonstrated by heat map analysis. Cluster analysis revealed that the hierarchical clustering of metabolites differed among rice varieties (**Figure 4A**, **Table S3**, **Sheet 5**). Principal component analysis (PCA) demonstrated that the variation in metabolites was mainly attributable to differences between rice varieties. Samples from the same rice variety were clustered together based on their similar metabolic profiles, thus supporting the stability and reliability of the subsequent analyses (**Figure 4B**).

After conducting quantitative analyses on all the detected metabolites and applying a fold change threshold, a total of 1,687 DAMs were identified between IR64-7d and ZH11-7d. Of these, 810 metabolites were upregulated in ZH11-7d, while 877 metabolites were downregulated (**Figure 4C**, **Table S3**, **Sheet 6**). Further analysis enabled the identification of 253 of these metabolites (**Table S3**, **Sheet 7**). According to KEGG enrichment analysis, it was found that the DAMs identified in ZH11-7d were mainly enriched in five pathways as compared to IR64-7d. These pathways include biosynthesis of secondary metabolites (ko01110), flavone and flavonol biosynthesis (ko00944), flavonoid biosynthesis (ko00941), the pentose phosphate pathway (ko00030), and stilbenoid, diarylheptanoid, and gingerol biosynthesis (ko00945) (P-value <0.05) (**Figure 4D**, **Table S3**, **Sheet 8**).

In our research, we identified metabolites enriched in the pentose phosphate pathway when comparing the ZH11-7d and IR64-7d groups. We observed an increase in the contents of gluconic acid (M197T94), D-glucose (M163T93_1), and gluconolactone (M179T93) in the ZH1 group (**Figure S5**, **Table S3**, **Sheet 9**). Additionally, our findings highlighted the significance of plant secondary metabolism in enhancing plant resistance. Among the various classes of plant secondary metabolism, aromatic compounds such as stilbenoid, diarylheptanoid, gingerol, and flavonoids were the most important. Our results are consistent with previous reports that suggested the elevated accumulation of flavonoids enhances the resistance of grapes and strawberries to *Botrytis cinerea* (Xu et al., 2019).

To summarize, the results showed that rice variety ZH11, when infected with *M. graminicola*, had an increase in DAMs related to the pentose phosphate pathway and secondary metabolism pathway, which helped to improve its resistance to *M. graminicola* infection as compared to rice variety IR64.

### Integrated transcriptome and metabolome analysis

**Figure 5:**
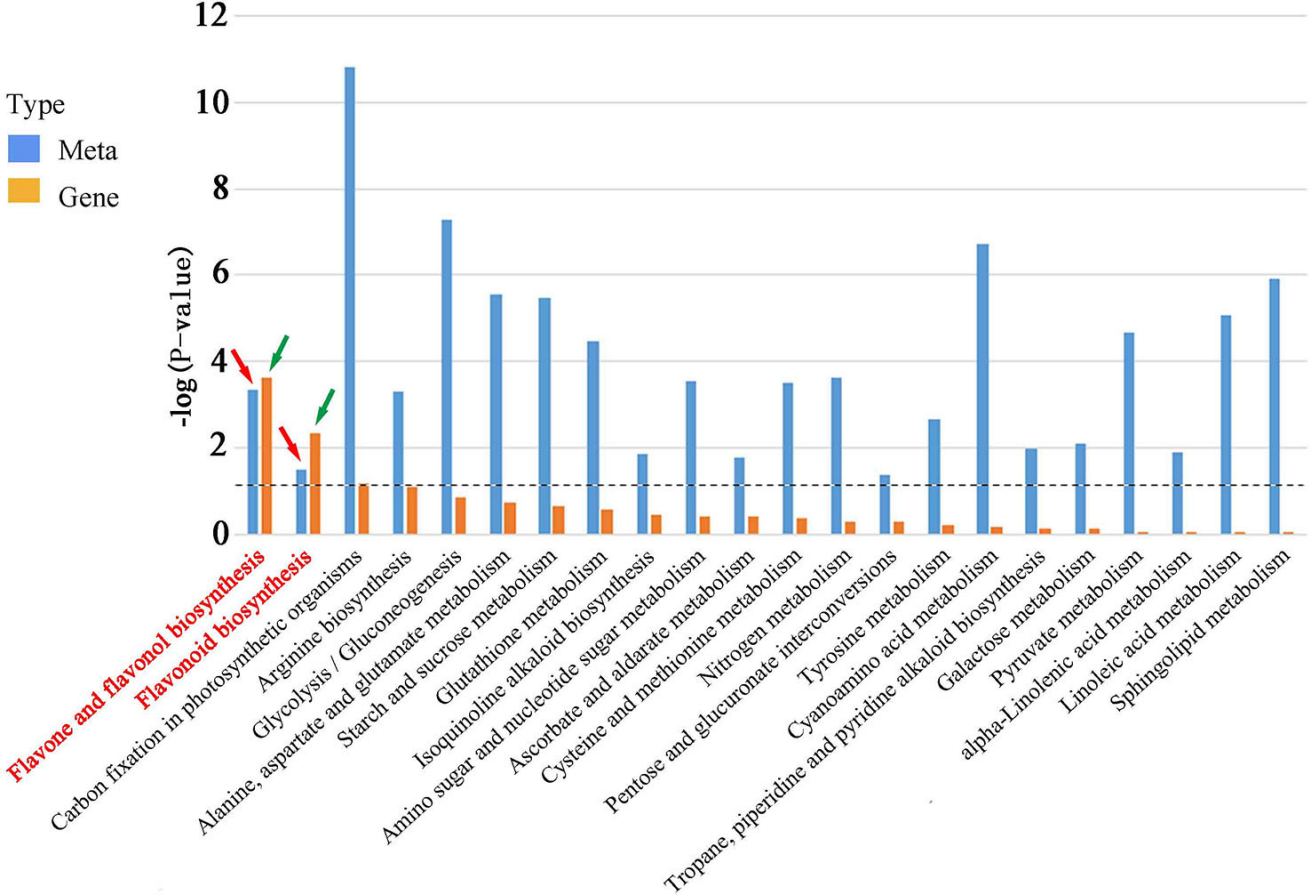
DEG and DAM enrichment in KEGG pathways. A P-value was calculated by a hypergeometric test, which indicates the degree of enrichment of DEGs or DAMs. The red arrow indicates metabolites enriched in flavone and flavonol biosynthesis (ko00944) and flavonoid biosynthesis (ko00941), and the green arrow indicates genes enriched in flavone and flavonol biosynthesis (ko00944) and flavonoid biosynthesis (ko00941). The dotted line indicates the size or column height when the p-value is 0.05.

To investigate the correlation between DEGs and DAMs in rice roots during *M. graminicola* infection, a comprehensive analysis of the transcriptome and metabolome was carried out. The KEGG enrichment analysis revealed that 23 pathways were co-enriched in both DAMs and DEGs. Notably, two of these pathways, flavone and flavonol biosynthesis (ko00944) and flavonoid biosynthesis (ko00941), were found to be highly significant (p-value <0.05). These results indicate that flavonoids play a crucial role in enhancing rice resistance to *M. graminicola* infection (**Figure 5**, **Figure S6**, **Table S3**, **Sheet 10**).

**Figure 6:**
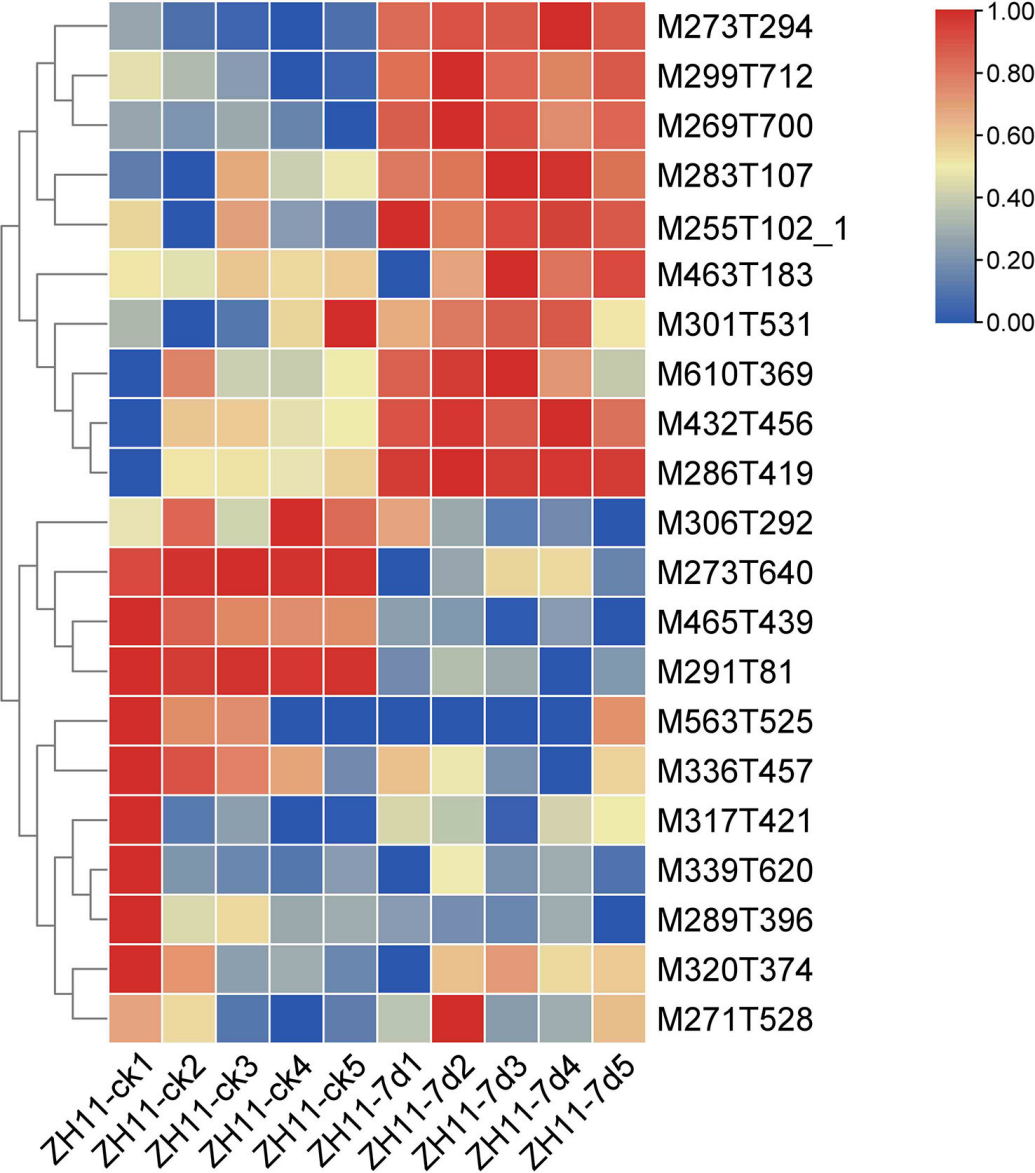
The heatmap shows the content of compounds involved in flavonoids metabolism in healthy roots (ZH11-ck) and infected roots (ZH11-7d) of rice ZH11. Metabolites with increased content during nematode infection were M273T294 (Apiforol), M299T712 (Kaempferide), M269T700 (Apigenin), M283T107 (Quercetin), M255T102_1 ((S)-Pinocembrin), M463T183 (Isoquercitrin), M301T531 (Hesperetin), M610T369 (Rutin), M432T456 (Apigenin 7-O-beta-D-glucoside), M286T419 (Kaempferol). In different samples, the metabolic compounds showed similar states for Pearson clustering.

In this study, analysis of the rice ZH11 metabolome data revealed a significant increase in the levels of ten flavonoids upon infection with *M. graminicola*. Specifically, these flavonoids were identified as M273T294 (Apiforol), M299T712 (Kaempferide), M269T700 (Apigenin), M283T107 (Quercetin), M255T102_1 ((S)-Pinocembrin), M463T183 (Isoquercitrin), M301T531 (Hesperetin), M610T369 (Rutin), M432T456 (Apigenin 7-O-beta-D-glucoside), and M286T419 (Kaempferol) (**Figure 6**, **Table S4**, **Sheets 1, 2**). Of particular interest were Kaempferide, Apigenin, and Quercetin, as previous research has demonstrated their potential to enhance plant resistance. Especially, Kaempferide has been shown to exhibit resistance to *Fusarium oxysporum*, while Apigenin has been found to enhance resistance to aphids. Quercetin, on the other hand, is a potent antioxidant that has been shown to improve plant tolerance (Curir et al., 2001; Singh et al., 2021; Zhou et al., 2021).

**Figure 7:**
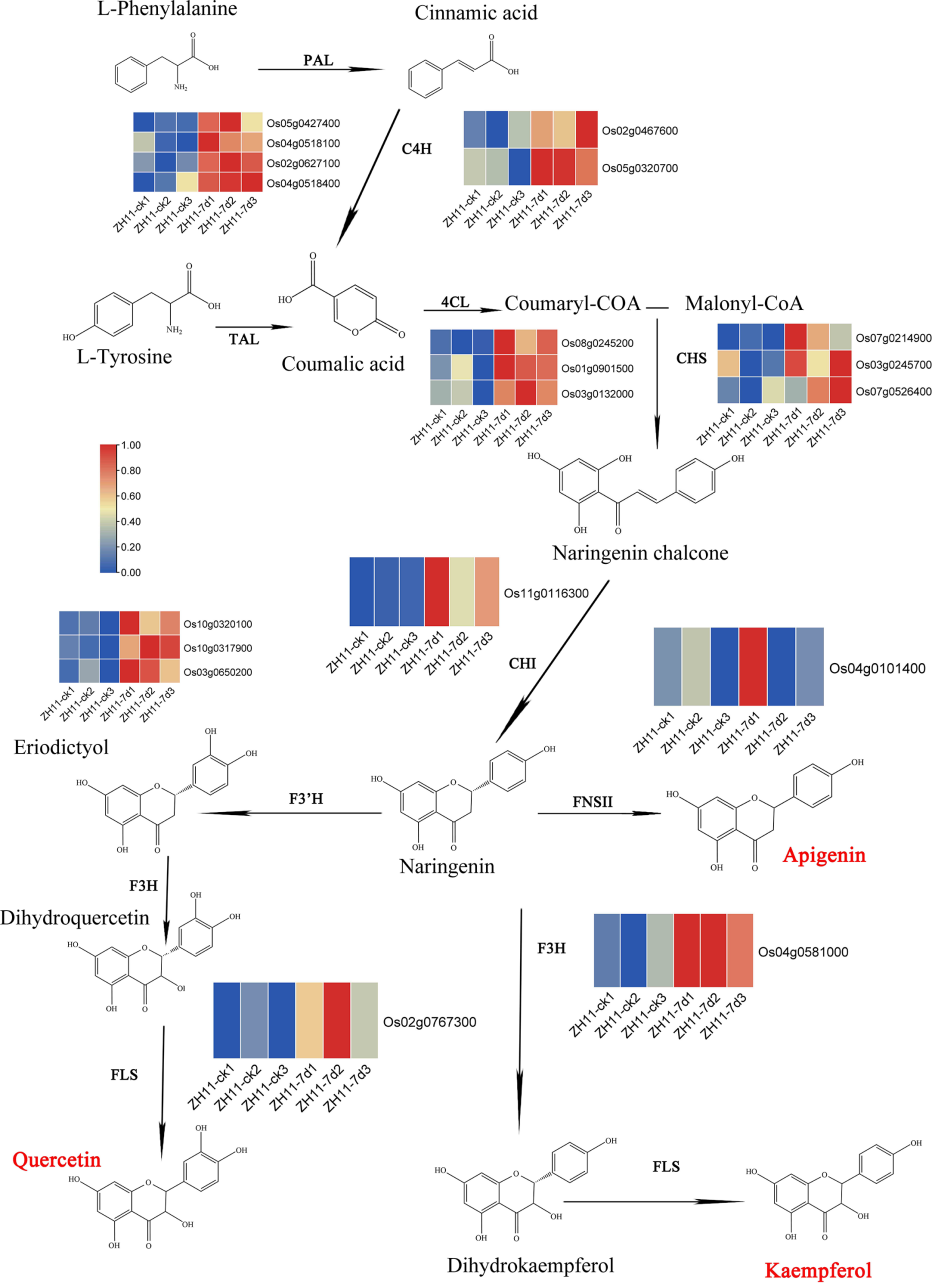
The metabolic pathway of flavonoids and the expression heatmap of the genes involved in this pathway from *O. sativa* infected by *M. graminicola.* PAL: phenylalanine ammonia lyase (*Os05g0427400*, *Os04g0518100*, *Os02g0627100* and *Os04g0518400*); C4H: cinnamate 4 hydroxylase (*Os02g0467600* and *Os05g0320700*); TAL: tyrosine ammonia lyase; 4CL: coumaric acid COA ligase (*Os08g0245200*, *Os01g0901500* and *Os03g0132000*); CHS: chalcone synthetase (*Os07g0214900*, *Os03g0245700* and *Os07g0526400*); CHI: chalcone isomerase (*Os11g0116300*); FNSII: flavone synthase II (*Os04g0101400*); F3’H: flavonoid3’-hydroxylasc (*Os10g0320100*, *Os10g0317900* and *Os03g0650200*); F3H: flavanone-3-hydroxylase (*Os04g0581000*); FLS: flavonol synthase (*Os02g0767300*).

We further summarized the synthetic pathway of these three flavonoids and identified the related genes involved in the metabolic pathways. Our analysis revealed a total of 76 genes participating in flavonoid metabolism within the rice genome (**Figure 7**, **Table S4**, **Sheet 3**). Further investigation revealed that, out of the 76 genes, 25 were upregulated in the comparison group ZH11-7d vs ZH11-ck. However, one gene was downregulated in the comparison group IR64-7d vs IR64-ck (**Figure S7**). Out of the 25 upregulated genes, 19 genes were identified as playing a role in the synthesis of Kaempferide, Apigenin, and Quercetin. These genes included *Os05g0427400*, *Os04g0518100*, *Os02g0627100*, and *Os04g0518400*, which encode for PAL (phenylalanine ammonia lyase); *Os02g0467600* and *Os05g0320700*, which encode for C4H (cinnamate 4 hydroxylase); *Os08g0245200*, *Os01g0901500*, and *Os03g0132000*, which encode for 4CL (coumaric acid COA ligase); *Os07g0214900*, *Os03g0245700*, and *Os07g0526400*, which encode for CHS (chalcone synthetase); *Os11g0116300*, which encodes for CHI (chalcone isomerase); *Os04g0101400*, which encodes for FNSII (flavone synthase II); *Os10g0320100*, *Os10g0317900*, and *Os03g0650200*, which encode for F3’H (flavonoid-3’-hydroxylase); *Os04g0581000*, which encodes for F3H (flavanone-3-hydroxylase); and *Os02g0767300*, which encodes for FLS (flavonol synthase) (**Table S4**, **Sheet 4**). The heat maps of transcriptome data indicate that the expression of these 19 genes was upregulated during *M. graminicola* infection in ZH11 root tissues (**Figure 7**).

These findings suggest that flavonoids play a crucial role in rice disease resistance and are actively involved in the response to *M. graminicola* infection.

### Verification of the expression of genes involved in flavonoid biosynthesis pathways

**Figure 8:**
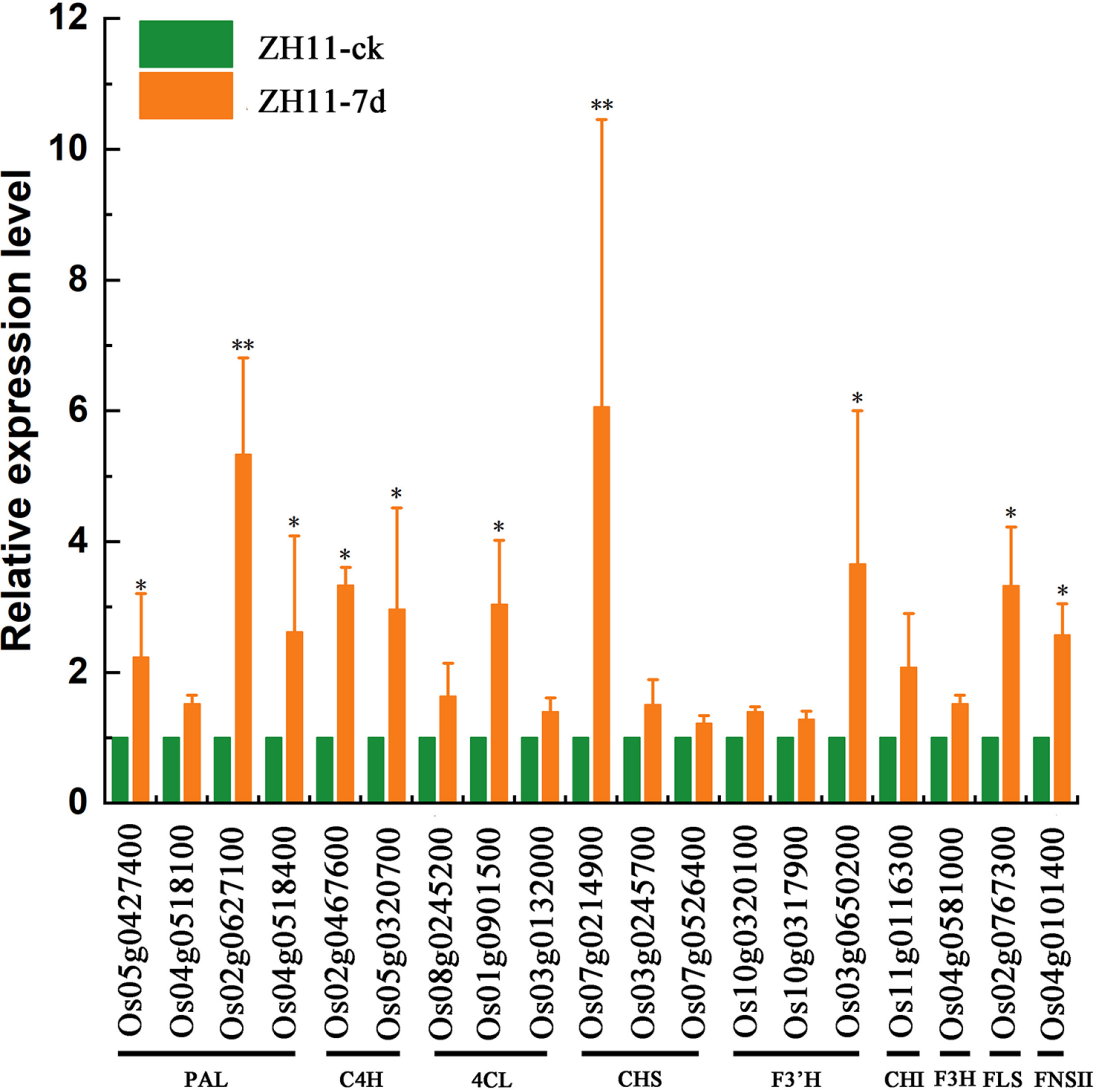
The relative expression of genes involved in the flavonoid biosynthesis in ZH11 roots infected by *M. graminicola.* Expression level was measured by RT-qPCR and the means ± SE from three independent tests were calculated. Asterisks represent significant differences for the defense-related genes using Duncan’s test. (*p <0.05, **p <0.01).

We confirmed alterations in the expression of genes related to flavonoid biosynthesis pathways in the root tissues of rice varieties ZH11 and IR64, following infection by *M. graminicola*, using RT-qPCR. The results demonstrated a significant upregulation (P-value <0.05) of PAL encoding genes *Os05g0427400*, *Os02g0627100*, and *Os04g0518400*, C4H encoding genes *Os02g0467600* and *Os05g0320700*, 4CL encoding gene *Os01g0901500*, CHS encoding gene *Os07g0214900*, F3’H encoding gene *Os03g0650200*, FLS encoding gene *Os02g0767300*, and FNSII encoding gene *Os04g0101400* in rice variety ZH11 when infected by *M. graminicola*. This indicated that the synthesis of Kaempferide, Apigenin, and Quercetin was increased, thereby enhancing the resistance of ZH11 against *M. graminicola* (**Figure 8**). Despite the nematode infection, the expression levels of 19 genes related to flavonoid biosynthesis in IR64 root tissues did not increase. Instead, some of these genes showed a downward trend, indicating that the infection may be hindering the disease resistance response of rice variety IR64 in some manner (**Figure S8**).

## Discussions

Maintaining stable production of rice is crucial for ensuring the survival of millions of people around the world since it is the largest food crop. However, the occurrence of various diseases and insect pests in rice agricultural production significantly limits its yield and quality. Examples of such diseases and pests include rice blast disease, rice stem borer, and nematode disease (Skamnioti and Gurr, 2009; Reagan and Mulcahy, 2019). In particular, the occurrence of the rice root knot nematode, *M. graminicola*, has become increasingly severe in the primary rice-producing regions of China, leading to significant food loss (Ju et al., 2020; Luo et al., 2020; Liu et al., 2021). Therefore, it is essential to prevent and control the occurrence of *M. graminicola* in agricultural production.

Planting resistant varieties is the most efficient and eco-friendly method to prevent and manage nematode diseases. However, as there have been no reports on cloning major genes from rice for the resistant *M. graminicola* (Phan et al., 2018), exploring internal resistance genes to improve rice resistance would be more significant (Galeng-Lawilao et al., 2018). To this end, in this study, we conducted transcriptome and metabolome analyses on ZH11 (resistant) and IR64 (susceptible) rice plants infected with *M. graminicola*. Our results showed that ZH11 displayed significantly higher resistance to *M. graminicola* infection compared to IR64. Transcriptome analysis revealed that 3,765 genes were upregulated in ZH11, which far exceeded the 218 upregulated genes in IR64 in response to nematode infection. KEGG and GO analysis demonstrated that the upregulated genes in ZH11 were mainly involved in cell wall organization, which increased the toughness of rice plants and effectively countered nematode infection. Moreover, the metabolome analysis indicated significant enrichment of flavonoid metabolism in rice cells, highlighting the essential role of flavonoids in ZH11’s resistance to nematodes.

Flavonoids are a type of secondary metabolite found in plants that serve as a defense mechanism against microorganisms. Research indicates that these compounds can affect nematode egg survival, fecundity, and attraction to host roots (Grundler et al., 1991; Wuyts et al., 2006). When plant roots are infected with nematodes, flavonoids are induced, and resistant plants have been shown to have higher concentrations of flavonoids compared to susceptible plant varieties (Li et al., 2021). In particular, specific flavonoids are induced at the feeding site during the interaction between plants and nematodes, which suggests that resistant plants are better equipped to accumulate flavonoids to combat disease compared to susceptible plants (Grundler et al., 1991). However, it remains unclear why the induction of similar chemical reactions in susceptible plants is not successful when they are infected with nematodes. Therefore, the question remains: how are flavonoids induced in disease-resistant plants when they are attacked by nematodes?

We are aware that the synthesis of plant flavonoids is regulated by various transcription factors, such as the MYB–bHLH–WDR protein complex (Xu et al., 2015). For example, MYBF1 combined with the CHS promoter regulates flavonol synthesis in citrus and grape (Comeskey et al., 2009; Liu et al., 2016), while PabHLH1 plays a role in flavonoid and dibenzyl compound biosynthesis in liverworts and stimulates flavonol and anthocyanin accumulation in Arabidopsis (Zhao et al., 2019). Additionally, TTG1 forms a complex with MYB and BHLH to participate in flavonoid and anthocyanin secondary metabolism in plants, improving their resistance to pathogens (Miller et al., 2015). Based on this knowledge, we hypothesize that the activity of certain transcription factors may be induced by *M. graminicola* infection in rice, which could trigger flavonoid metabolism, increase flavonoid content, and enhance rice’s disease resistance. Therefore, we aim to investigate the changes in transcription factor activity in ZH11 following *M. graminicola* infection and elucidate the mechanism behind rice’s disease resistance in future experiments.

Currently, research on *M. graminicola* and rice interactions has primarily focused on the mechanisms used by *M. graminicola* to evade the rice immune system. For instance, Chen et al. (2017) discovered that MgGPP, a secreted factor, infiltrates rice cells and targets the endoplasmic reticulum, using the host cells to carry out glycosylation modification, which inhibits the rice immune response (Chen et al., 2017). Similarly, Song et al. (2021) found that the effector MgMO289 targets a new copper chaperone, metallochaperone, to eliminate O_2_•^−^ using the host O_2_•^−^ scavenging system, thus inhibiting plant immunity (Song et al., 2021). Additionally, Chen et al. (2018) revealed that the effector MgMO237 inhibits the host defense response by interacting with various host defense pathways, including the expression of defense-related genes, callose deposition on the cell wall, and the outbreak of reactive oxygen species, thereby promoting parasitism in rice (Chen et al., 2018). These studies suggest that *M. graminicola* utilizes effectors to evade the rice immune response, enabling parasitism and pathogenicity in rice cells. Moreover, the reports mentioned above highlight the existence of disease-resistant proteins in rice ZH11 that can interact with effectors secreted by *M. graminicola* and hinder their inhibition of rice disease resistance. These resistance proteins can activate a series of signals, such as salicylic acid and jasmonic acid accumulation, reactive oxygen species outbreak, secondary metabolite synthesis, and lignin and callose accumulation, which enhance the resistance of ZH11. However, the research has yet to report on these disease-resistant proteins, which represents an important area for future research.

In the following research, we need to place greater emphasis on examining the interaction between rice and *M. graminicola*. Specifically, we should investigate how nematodes are able to evade the immune response of rice and how rice recognizes the presence of nematode infection and activates defense responses. Of particular importance in this study is analyzing the role of flavonoids in the resistance pathway and connecting them to the rice resistance pathways. This will provide valuable theoretical guidance in enhancing our understanding of the rice resistance mechanism.

## Materials and methods

### Plant material and growth conditions

The rice varieties used in this experiment were ZH11 (a disease-resistant variety) and IR64 (a disease-susceptible variety) procured from our laboratory. The seeds of ZH11 and IR64 were germinated for 4 days at 30°C in water; the saplings were then transferred for planting. Three seedlings were planted in a plastic flowerpot with an opening diameter of 10 cm, a bottom diameter of 7.5 cm, and a height of 8.5 cm. The flowerpot was filled with nutrient soil and placed in an incubator for rice cultivation. The incubation conditions were 25°C room temperature, 75% relative humidity, a 12-hour light and dark period each day, and adequate light intensity to meet the natural growth requirements of rice.

### Analysis of symptoms in *O. sativa* following nematode inoculation

We raised the experimental nematode, *M. graminicola*, in our laboratory. To obtain the nematode suspension, we rinsed rice roots infected with the nematodes under water, broke the root knots using tweezers, and cut the roots. We then saved the roots in a paper towel, soaked the paper towel in a petri dish containing water, and collected the nematode suspension after 1–2 days. The rice seedlings were transplanted into nutrient soil and inoculated with *M. graminicola* 15 days later. We inoculated each plant with 500 nematodes by introducing the nematode suspension into the rice roots. The inoculated rice was returned to the incubator for further culture. After some time, we observed white tips on the rice leaves, which were indicative of *M. graminicola* infection.

### Plant material and RNA preparation

In the present study, we collected roots from two rice varieties, ZH11 (disease resistant) and IR64 (disease susceptible), seven days after infection with *M. graminicola* as described in previous reports (Kyndt et al., 2012). Untreated rice roots were used as a control. These samples were grouped into IR64-ck, IR64-7d, ZH11-ck, and ZH11-7d, each consisting of three biological replicates. Total RNA was extracted from each group using the Plant/Fungi RNA Purification Kit from Sigma-Aldrich Trading Co. Ltd. (Shanghai, China), and the quality of the RNA was assessed using a Germany IMPLEN P330 ultra-microspectrophotometer. Only RNA of high quality was utilized for library preparations using the TruSeq SBS Kit (Illumina Inc., San Diego, CA, USA) and paired-end sequencing on an Illumina Hi-Seq 2000 platform (Illumina) at Suzhou PANOMIX Biomedical Tech Co., Ltd. (Suzhou, China).

### Quality analysis of transcriptome data

The genome of *Oryza sativa* Japonica was obtained from the Ensembl plant (http://plants.ensembl.org/) and underwent a quality control process using Trimmomatic to remove low-quality sequences to obtain clean reads (Bolger et al., 2014). The clean reads were then aligned to the *O. sativa* genome using hisat2 software (version: 2.0.1-beta) (version: 2.0.1-beta) (Kim et al., 2019), and the alignment quality was evaluated based on Q20, Q30, and GC content. For transcriptome sequencing, root tissues were collected from 12 samples belonging to four groups of rice, namely IR64-ck, IR64-7d, ZH11-ck, and ZH11-7d. The Illumina Hi-Seq 2000 platform was used for sequencing, and transcriptome reconstruction was performed using Stringtie (version 2.0.4) and Gffcompare software (Pertea et al., 2015; Pertea and Pertea, 2020). The size of the transcriptome data for each of the 12 samples ranged from 6.2 Gb to 7.0 Gb. After removing low-quality reads and adaptor sequences from the raw data, the high-quality clean reads were between 5.7 Gb and 6.5 Gb. Furthermore, the Q20 of clean reads ranged from 96.64% to 97.32%, and the Q30 ranged from 92.07% to 93.06% (**Table S1**, **Sheet 1**).

The clean reads were filtered and aligned to the *O. sativa* genome using hisat2 software (Kim et al., 2019). The results revealed that at least 83.15% and up to 94.27% of the reads were successfully mapped in the 12 samples, with over 93% of them being unique (**Table S1**, **Sheet 2**). Moreover, the distribution of reads across the genome was examined, and the regions were categorized into gene (ORF), intergene (intron regions), and exon regions. The findings showed that more than 93.74% of the mapped reads in the 12 samples were localized to the effective gene regions of the *O. sativa* genome (**Table S1**, **Sheet 3**).

### Analysis of gene expression among 12 samples

After obtaining the effective reads, we utilized featureCounts software (version: v1.6.0) to determine the number of mapped reads for the genes based on the *O. sativa* genome annotation file (Liao et al., 2014). The FPKM (fragments per kilobase of exon per million reads mapped) method was standardized for gene expression analysis (**Table S1**, **Sheet 4**) (Zhao et al., 2021). The principal component analysis (PCA) revealed that samples from the same plant were grouped together (**Figure S1**). The heat map indicated that gene expression was similar within the same samples but differed significantly among different samples (**Figure S2**).

We employed the R package edgeR to analyze the gene expression differences among the various samples (Robinson et al., 2010). We used a threshold of a P-value less than 0.05 and a FoldChange absolute value greater than 2 to identify significantly differentially expressed genes (DEGs). The DEGs among the different samples are presented in **Table S2**, **Sheets 1–4**. To gain insights into the function of these DEGs, we performed GO and KEGG analyses using TBtools (Chen et al., 2020). The results of the GO and KEGG enrichment analyses are presented in **Tables S2**, **S3**.

### Data availability

RNA-Seq data described in this paper have been submitted to the Genome Sequence Archive at the National Genomics Data Center (NGDC) (https://ngdc.cncb.ac.cn/), accession number CRA009084.

The data reported in this paper have been deposited in OMIX, China National Center for Bioinformation/Beijing Institute of Genomics, Chinese Academy of Sciences (https://ngdc.cncb.ac.cn/omix; accession number: OMIX00248).

### Widely targeted metabolome analysis

The rice roots of ZH11 and IR64 infected by *M. graminicola* on the 7 days, marked as ZH11-7d and IR64-7d, were collected for the metabolome analysis. The method of sample collection was the same as for the transcriptome analysis experiment, and three biological replicates were carried out for each sample. Metabolome analysis was conducted by Suzhou PANOMIX Biomedical Tech Co., Ltd. (Suzhou, China). (1) Accurately weigh 93.6–200.8 mg ( ± 1%) of rice root tissues in a 2 ml EP tube, add 0.6 ml 2-chlorophenylalanine (4 ppm) methanol (−20 °C), and vortex for 30 s (when the sample size was less than or equal to 100 mg, the extraction system was halved); (2) Add 100 mg of glass beads, place it on a tissue grinder, and grind for 90 s at 60 Hz; (3) sonicate for 15 min at room temperature; (4) centrifugation at 12,000 rpm at 4 °C for 10 min, take out 300 μl of the supernatant, filter through a 0.22 μm membrane, and add the filtrate to the detection bottle; (5) take 20 μl from each sample for the quality control (QC) samples (these QC samples were used to monitor deviations of the analytical results from the pool mixtures and compare them to observe the errors caused by the analytical instrument itself); (6) use the rest of the samples for LC-MS detection. All the results are shown in **Table S3**, **Sheet 5**.

Differentially accumulated metabolites (DAMs) were screened out based on the variable importance of projection (VIP) ≥1 and |log2(foldchange)|≥ 1 (**Table S3**, **Sheets 6, 7**). The functions of DAMs were further analyzed using the KEGG compound database to determine the metabolic pathways that were most highly correlated with the resistance of *O. sativa* to *M. graminicola* infection (**Table S3**, **Sheet 8**).

### Quantitative real-time PCR analysis

The RNA was extracted from the roots of ZH11-ck and ZH11-7d using the Plant/Fungi RNA Purification Kit from Sigma-Aldrich Trading Co. Ltd. (Shanghai, China). The quantity and quality of RNA were checked and measured using a Germany IMPLEN P330 ultra-microspectrophotometer. We synthesized cDNA with *EasyScript* ^®^ One-Step gDNA Removal and cDNA Synthesis SuperMix (TRAN) according to the manufacturer’s instructions. cDNA was used to detect gene expression by quantitative real-time PCR (RT-qPCR). RT-qPCR was performed on a CFX 96 real-time PCR detection system (Bio Rad). PCR was performed with *PerfectStart* Green qPCR SuperMix^®^ (TRAN). PCR results were analyzed using the 2^−ΔΔCt^ method (Livak and Schmittgen, 2001). All biological experiments were repeated three times for each sample. The RT-qPCR primer sequences used are listed in **Table S5**.

## Data availability statement

The datasets presented in this study can be found in online repositories. The names of the repository/repositories and accession number(s) can be found in the article/**Supplementary Material**.

## Author contributions

XGS, XTS, WK and RC conceived and designed the experiments. LZ, SL, CS, YKL, YZ, LY and YCL performed the experiments. GX and JM analyzed the data. LZ, MA, XGS, XTS, WK and RC wrote the paper. All authors contributed to the article and approved the submitted version.
