# Supplementary material for: Integrated transcriptome and metabolome analysis revealed that flavonoids enhanced the resistance of Oryza sativa against Meloidogyne graminicola
Source: Front Plant Sci. 2023 Mar 31;14:1137299. doi: 10.3389/fpls.2023.1137299 (PMC10102519; doi:10.3389/fpls.2023.1137299)
Supplement: Supplementary file 6 [file DataSheet_1.docx]

***Supplementary Figures***


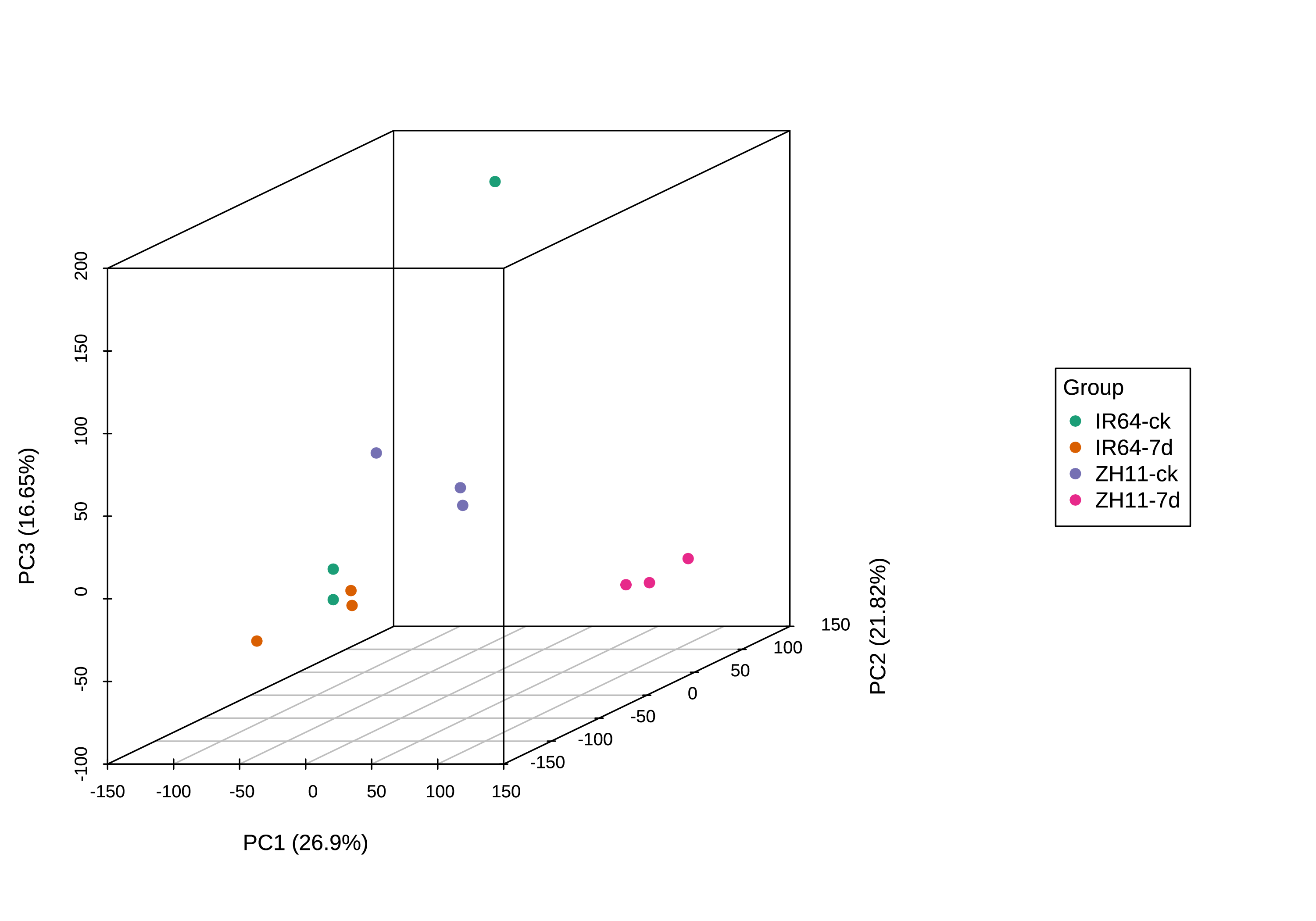


Fig.S1 The gene expression profiles of 12 samples were analyzed by PCA.


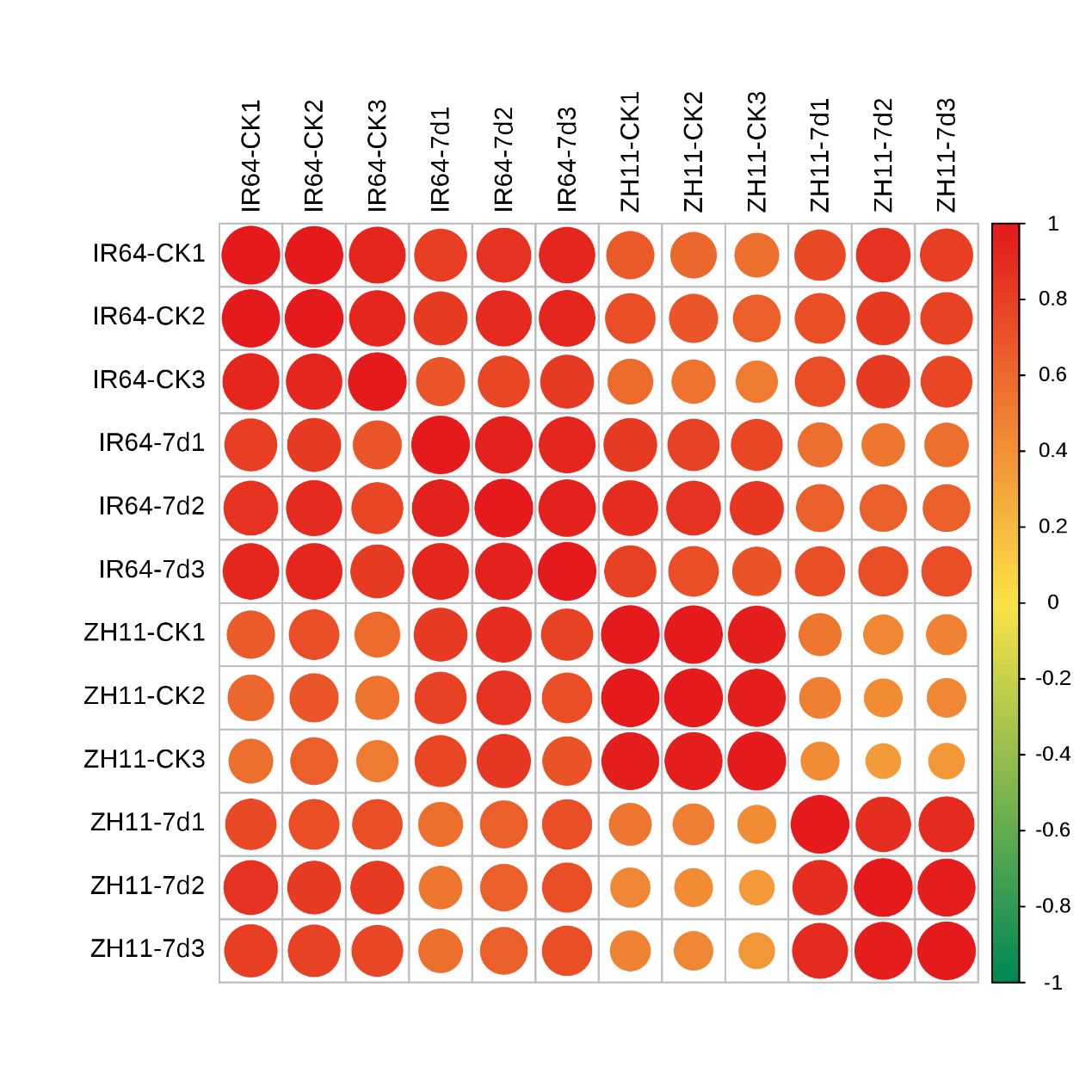


Fig.S2 The correlation analysis of gene expression levels among samples showed that the samples had a high consistency in one group.


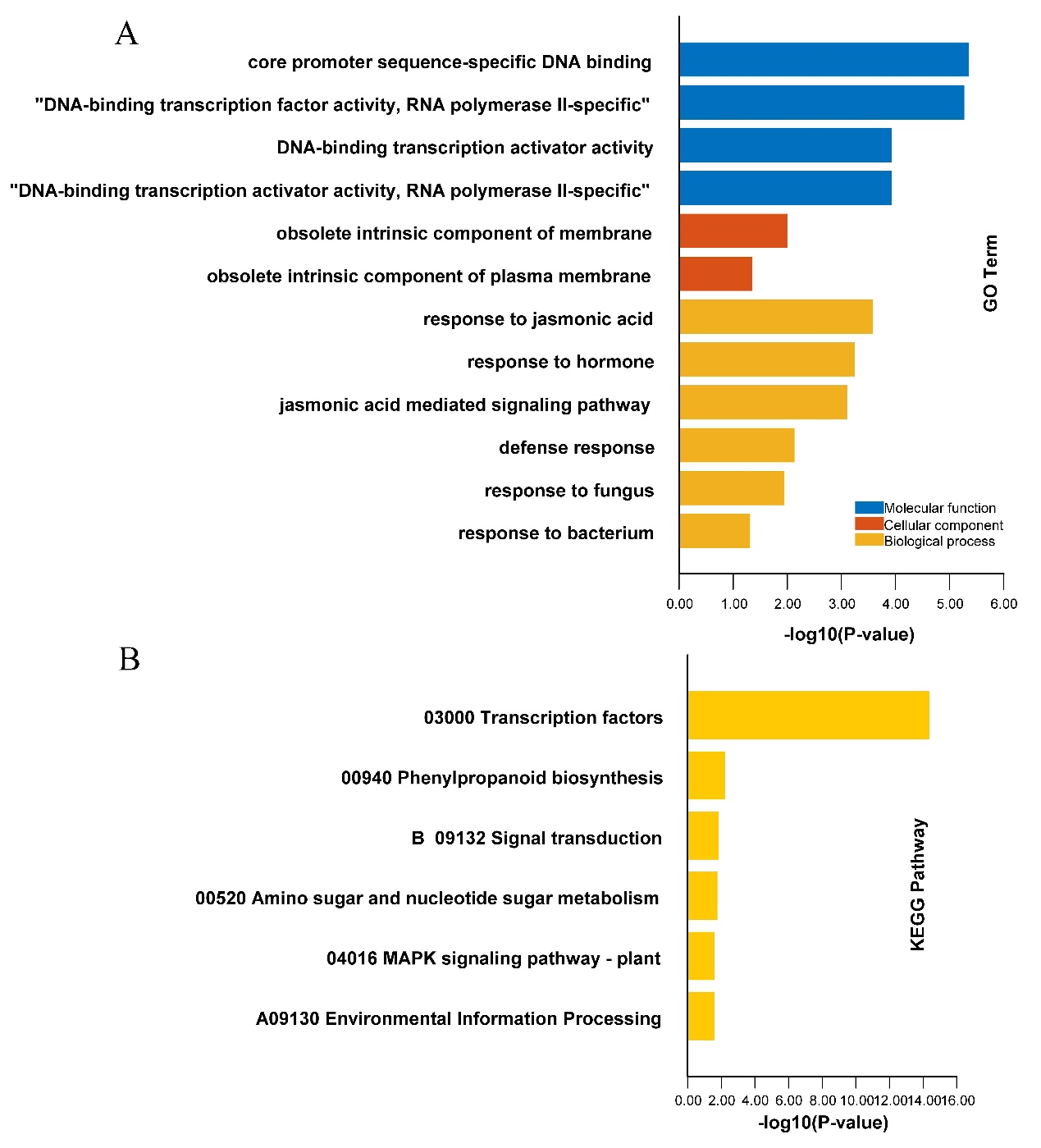


Fig.S3 GO and KEGG analysis of DEGs in comparison group IR64-7d vs IR64-ck. A indicated that the GO enrichment of DEGs in comparison group IR64-7d vs IR64-ck. B indicated that the KEGG enrichment of DEGs in comparison group IR64-7d vs IR64-ck.


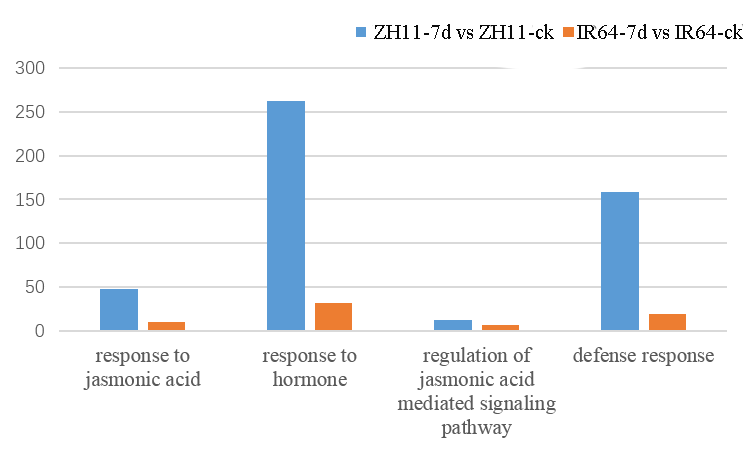


Fig. S4 The number of genes from comparison group IR64-7d vs IR64-ck enriched in response to jasmonic acid (GO:0009753), response to hormone (GO:0009725), regulation of jasmonic acid mediated signaling pathway (GO:2000022) and defense response (GO:0006952) were significantly less than that genes from comparison group ZH11-7d vs ZH11-ck.


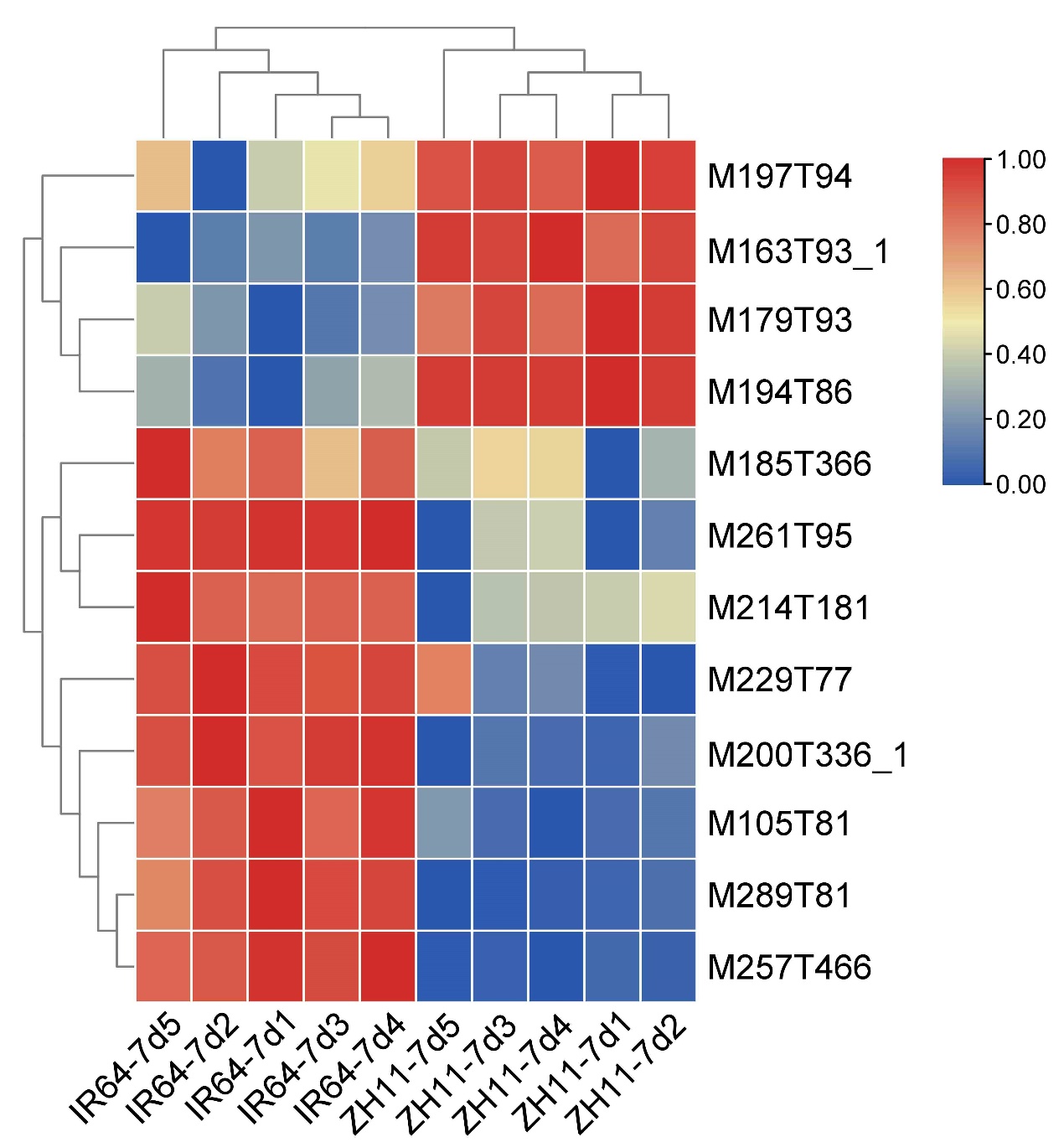


Fig. S5 Heat map analysis of various metabolites enriched in pentose phosphate pathway (ko00030). M229T77 represented D-Ribulose 5-phosphate; M257T466 represented 6-Phosphogluconic acid; M261T95 represented Beta-D-Glucose 6-phosphate; M197T94 represented Gluconic acid; M163T93_1 represented D-Glucose; M179T93 represented Gluconolactone; and M105T81 represented Glyceric acid (Table S3, Sheet9).


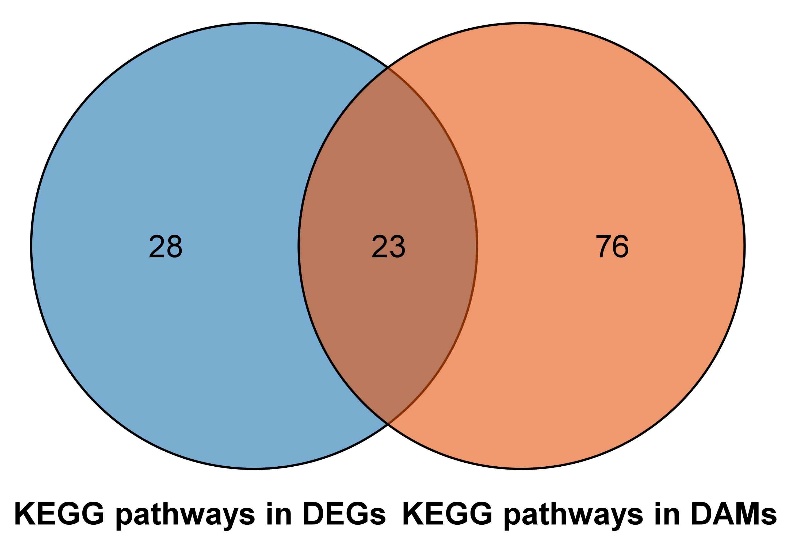


Fig.S6 Venn diagram showed the KEGG pathways co-enriched in DEGs and DAMs.


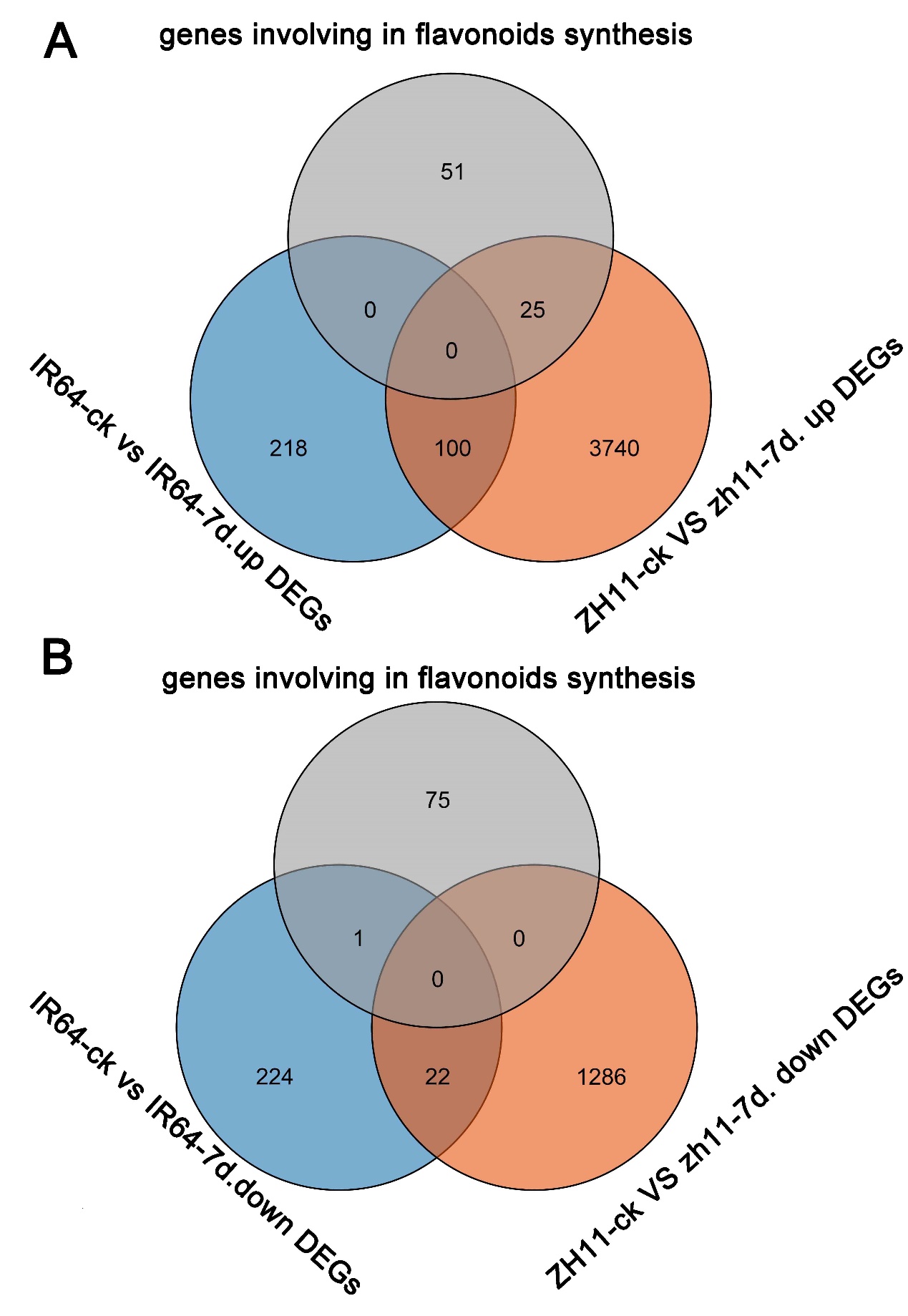


Fig.S7 Venn diagram showed the different expression genes involved in flavonoids synthesis in comparison group ZH11-7d vs ZH11-ck and IR64-7d vs IR64-ck*.* There were 25 genes up-regulated in comparison group ZH11-7d vs ZH11-ck, but no gene up-regulated in comparison group IR64-7d vs IR64-ck (A). While, there was one gene down-regulated in comparison group IR64-7d vs IR64-ck (B).


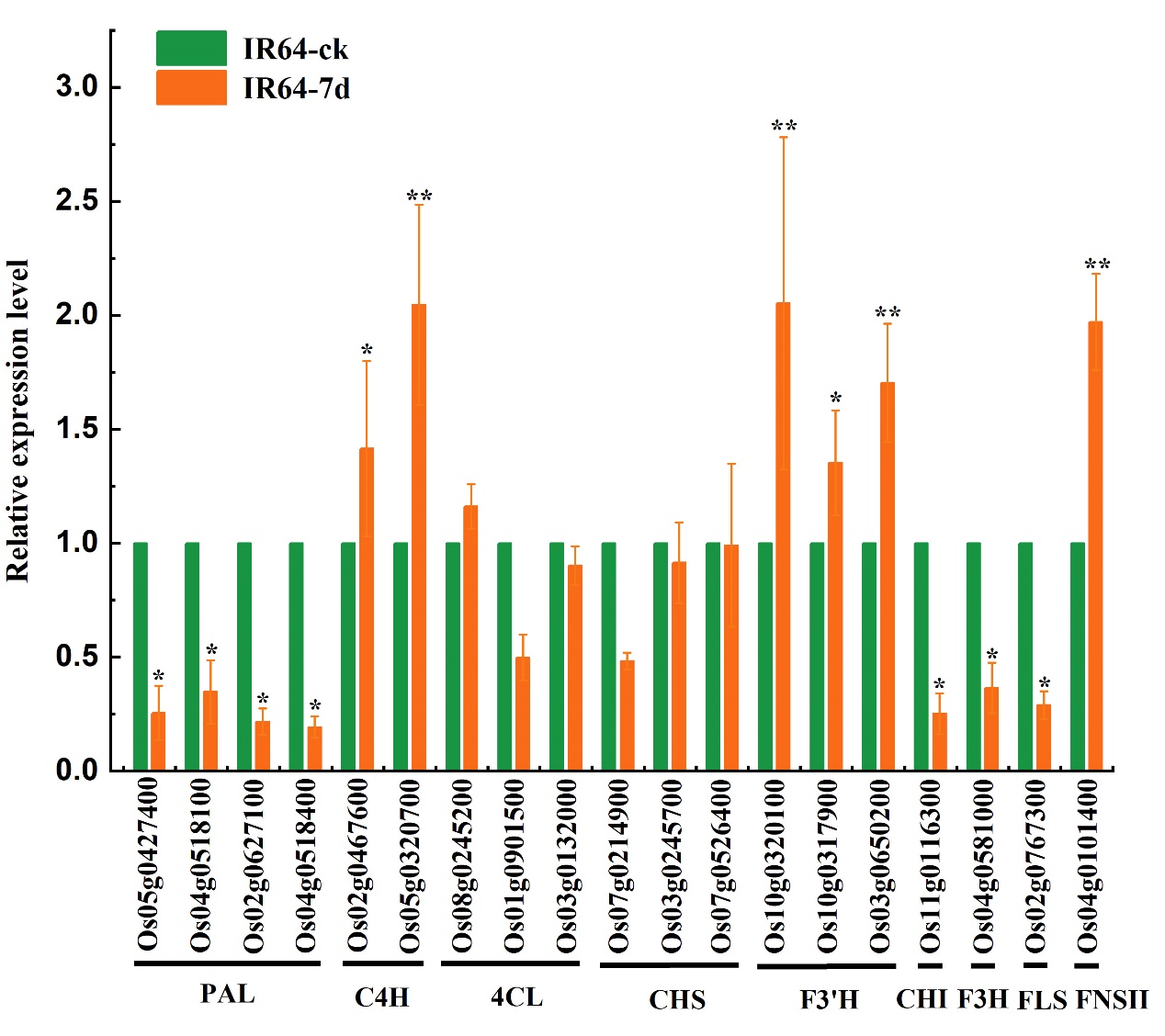


Fig.S8 The relative expression of genes involving in the flavonoids biosynthesis in IR64 roots infected by *M. graminicola.* Expression level were measured by RT-qPCR and the data were means of ±SE from three independent tests. Asterisks present significant difference for the defense-related genes using Duncan’s test. (**p* <0.05, ***p*<0.01).
